# Epidemiological and Clinical Profile of Hand, Foot, and Mouth Disease in Children in a Tertiary Care Center in Jammu

**DOI:** 10.7759/cureus.58704

**Published:** 2024-04-21

**Authors:** Aakriti Khajuria, Divyanshu Saini, Ravinder K Gupta, Amber Sharma, Sunny Babber

**Affiliations:** 1 Department of Pediatrics, Acharya Shri Chander College of Medical Sciences and Hospital, Jammu, IND; 2 Department of Community Medicine, Acharya Shri Chander College of Medical Sciences and Hospital, Jammu, IND

**Keywords:** vesiculopapular rash, hand-foot-and-mouth disease, enterovirus a-71, clinico-epidemiological profile, coxsackievirus a-16

## Abstract

Background

Hand, foot, and mouth disease (HFMD) is a viral illness commonly seen in children under five years of age, characterized by typical manifestations such as oral lesions and rashes on the hands and feet. Coxsackievirus A-16 (CV-A16) and Enterovirus A-71 (EV-A71) are the major etiological agents of this disease. Over the past two decades, there have been several outbreaks of HFMD all across India. As there is no chemoprophylaxis available for the disease, it becomes even more significant to conduct regular research and surveillance for HFMD.

Aim and objective

To observe the clinico-epidemiological profile along with constitutional symptoms in HFMD patients attending pediatric OPD.

Methods

This hospital-based prospective observational study was conducted in the Post Graduate Department of Pediatrics, Acharya Shri Chander College of Medical Sciences and Hospital (ASCOMS & H), Sidra, Jammu and Kashmir, India, over six months from April to September 2023. A total of 132 children with symptoms of HFMD visited the pediatric OPD. After using inclusive and exclusive criteria, we selected a sample size of 112 children with HFMD. The descriptive data were expressed in terms of percentages and proportions, and their graphical representation was done using MS Excel (Microsoft Corporation, Redmond, Washington, United States).

Results

Among the 112 HFMD patients examined, the highest peak was seen in August, followed by another one in September. Most of the cases were seen in the age group of zero to three years, and it was observed that there was a linear fall in the number of cases with the increase in age. Nearly 61% of cases were male, showing a slight male preponderance. Vesiculopapular rash on the hand and foot was the most common clinical characteristic, whereas painful deglutition was noted to be the most common constitutional symptom in HFMD patients. About 27% had a positive family history, and nail changes post-recovery were present in 1.79% of cases during their regular follow-ups.

Conclusions

This study reveals that HFMD cases surged in August and September, with a history of contact in one-fourth of cases. Disease is seen more commonly in children under three years of age, and the incidence of cases decreases with the increase in age. The illness is usually contagious and can spread quickly; therefore, more awareness programs should be done to educate parents and promote hygiene to prevent contact cases.

## Introduction

Hand, foot, and mouth disease (HFMD) is a viral illness that is commonly seen in children under five years of age. It is caused by viruses of the genus Enterovirus, which belong to the family Picornaviridae, including polio, enteric cytopathic human orphan (ECHO), Coxsackievirus, and Enteroviruses. The two most important causes are Coxsackievirus A16 and Enterovirus 71. HFMD occurs in outbreaks, particularly in preschool children. Transmission occurs either by direct contact with an infected patient or through fomites [1]. The onset of HFMD begins with a prodrome phase, which is characterized by a low-grade fever and sore throat. This phase is further followed by the development of ulcers and blisters on the posterior aspect of the oral cavity [2]. This is further followed by a vesiculopapular skin rash on the palms, soles, buttocks, knees, elbows, and genital area. Mainly, the cases occur in the summer and early autumn [3]. Usually, no treatment is needed; it follows a benign and self-limiting course, and the illness resolves quickly over four to five days. HFMD can sometimes also lead to certain complications, like the temporary loss of a fingernail or toenail. Such complications usually occur four days after the onset of the disease. Further severe and rare cardiac and neurological complications have also been observed during these outbreaks, like encephalitis, meningitis, polio-like paralysis, myocarditis, and respiratory distress syndrome [4]. Mortality is due to cardiorespiratory failure in severely affected children. The diagnosis of HFMD is clinical and requires differentiation from other similar illnesses that cause oral ulcers like aphthous ulcers, herpangina, chicken pox, herpetic gingivostomatitis, Kawasaki disease, toxic epidermal necrolysis (TEN), viral pharyngitis, and Rocky Mountain spotted fever [5]. Treatment of HFMD is usually symptomatic and includes analgesics and a soft diet. Doctors also recommend isolating affected children at home and preventing disease. Currently, there is no pharmacological intervention or vaccine available for HFMD [6]. Over the last two decades, our understanding of HFMD has greatly improved, and it has received significant attention. Keeping the substantial impact of HFMD in mind, this study was conducted to know the clinical and epidemiological characteristics of HFMD patients presenting in the pediatrics department of a tertiary care hospital setting in Jammu.

## Materials and methods

Study design

This hospital-based prospective observational study was conducted after getting approval from the Institute of Independent Ethical Committee with reference no. ASCOMS/IEC/2023/Meeting-1/02. It was conducted in the Postgraduate Department of Pediatrics from April 2023 to September 2023 at Acharya Shri Chander College of Medical Sciences and Hospital (ASCOMS &H) in Jammu, India, over a period of six months. A total of 132 children with symptoms of hand, foot, and mouth disease visited the pediatric OPD. We selected a sample size of 112 children with HFMD after employing the inclusion and exclusion criteria in accordance with which all clinically diagnosed cases of HFMD children from the age group of zero to 13 years were included in the study, and the children above 13 years of age were excluded from the study. Patients diagnosed with HFMD underwent comprehensive clinical examinations, and their clinical manifestations and constitutional symptoms were documented based on detailed histories obtained from their parents or guardians. Informed consent was obtained from the parents or guardians of all participating children. Regular follow-ups were conducted to monitor post-recovery outcomes, including the presence of nail changes. Monthly records were maintained regarding the incidence of HFMD cases, along with the distribution of patients by age and gender. Clinical characteristics, such as the presence of vesiculopapular lesions on specific body parts and associated constitutional symptoms, were documented.

Statistical analysis

Using appropriate tools, statistical analysis was performed to analyze the collected data and draw meaningful conclusions regarding the epidemiological and clinical profile of HFMD in children in the tertiary care center in Jammu. We analyzed descriptive data using percentages and proportions and utilized Microsoft Excel (Microsoft Corporation, Redmond, Washington, United States) for a graphical representation of the collected data.

## Results

**Figure 1:**
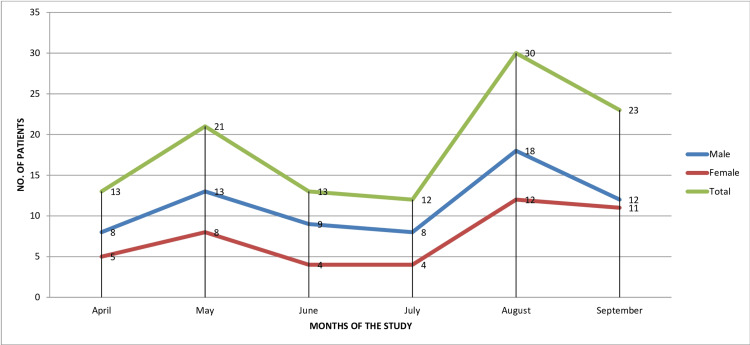
Month-wise incidence of HFMD cases according to gender (total cases, n=112) HMFD: hand, foot, and mouth disease

Figure 1 shows that out of the total 112 HFMD patients examined, the highest peak of cases was seen in August 2023 with n=30 cases (26.79%), followed by the second highest peak in September 2023 with n=23 cases (20.54%); however, minimum cases were observed in July 2023. Figure 1 also shows a slight male preponderance, as out of the total cases examined, n=68 cases (61%) were males and n=44 cases (39%) were females, with a male:female ratio of 1.54:1.

**Figure 2:**
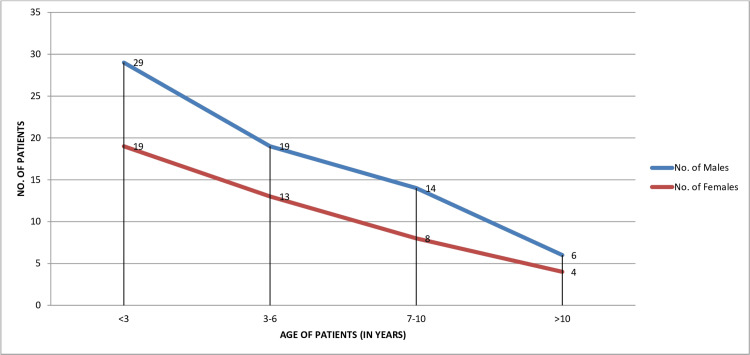
Distribution of HMFD cases according to their age and gender (total cases, n=112) HMFD: hand, foot, and mouth disease

Figure 2 shows the sex distribution with respect to the age groups. We divided the cases into four groups according to age: less than three years, three to six years, seven to 10 years, and more than 10 years. The highest number of cases, n=48 (42.86%), were seen in the age group of less than three years, and the lowest number of cases, n=10 (8.93%), were seen in the age group of more than 10 years. Additionally, we also observed that as the age increases, there is a linear fall in the number of cases.

Table 1 shows the various clinical characteristics seen in HFMD cases. In this study, we observed that 100% of the cases had vesiculopapular lesions on hands or feet, while vesiculopapular lesions on buttocks and elbows were seen in 91.96% and 48.21%, respectively. About 63% of the cases had oral lesions in the form of aphthous ulcers seen on the labial, palatal, buccal, and tongue regions, whereas 12.50% had oral lesions in the form of stomatitis.

**Table 1:** Clinical characteristics of HMFD cases (total cases, n=112 ) HMFD: hand, foot, and mouth disease

Clinical characteristics of lesions	No. of cases	Percentage (%)
Vesiculopapular lesions on hands or foot	112	100
Vesiculopapular lesions on buttocks	103	91.96
Vesiculopapular lesions on the elbow	54	48.21
Oral lesions in the form of aphthous ulcers	71	63.39
Oral lesions in the form of stomatitis	14	12.50

Table 2 shows the various constitutional symptoms seen among HFMD cases, where the most common symptom was painful deglutition, which was seen in 63.39% of cases, and the least common was dehydration, which was seen only in 7.14% of cases.

**Table 2:** Constitutional symptoms seen in HMFD cases (total cases, n=112) HFMD: hand, foot, and mouth disease

Associated symptoms	No. of cases	Percentage (%)
Painful deglutition	71	63.39
Fever	64	57.14
Loss of appetite	48	42.86
Lethargy and irritability	47	41.96
Pruritus	24	21.43
Sore throat	11	9.82
Dehydration	8	7.14

In this study, we also observed that in n=31 cases (27.68%), a history of contact was seen where the disease was present in another child of the same family currently or had HFMD infection in the past, suggesting the potential for person-to-person transmission within close contacts. Additionally, nail changes in the form of onychomadesis, Beau's lines, and yellowish-orange discoloration of the nail were observed in a few cases n=2 (1.79%) during follow-ups, highlighting potential post-recovery complications associated with HFMD.

## Discussion

Hand, foot, and mouth disease primarily occurs in infants and children and is occasionally seen in adults as well. It usually presents as mucocutaneous lesions and vesiculopapular rash on the hands, feet, buttocks, and oral cavity, as well as fever and upper respiratory symptoms, but it can sometimes lead to systemic complications. In most cases, the disease is self-limiting, and symptomatic treatment may be needed in outpatient care. Being contagious, it can spread through direct contact with the mucus, saliva, or faces of an infected person, leading to frequent outbreaks, especially during the summer and autumn months, and therefore has become an emerging public health problem. In India, over the last decade, major outbreaks were seen in the years 2004, 2007, 2012, and 2014, whereas the cases peaked in the years 2012 to 2015. In the union territory of Jammu and Kashmir in India, there was an outbreak of HFMD in May 2018, where 300 cases were reported [7]. The present study was also conducted in Jammu, in a tertiary care hospital, for a period of six months to see the current trend and clinical features along with constitutional symptoms seen in HFMD.

In the present study, we observed that 42.86% of cases were reported in children under three years of age. Another study done in Jammu (Udhampur) in 2023 by Monika Aggarwal [8] showed that 84% of cases were among children aged <5 years. Similarly, a study done in 2016 by Kumar [9] in Hyderabad, India, also showed more than 80% of cases to be below five years of age. These findings are also consistent with studies conducted in 2016 in China [10] and in 2021 in Singapore [11], where >70% of cases were seen in ages less than five years. The present study highlights that as age increases, there is a linear decrease in the number of HFMD cases, indicating a lower susceptibility among older children. A similar trend was seen in a study done in the year 2023 in Rajasthan, India [12]. Moreover, we observed a slight male predominance in HFMD cases with a male:female ratio of 1.5:1, which is in sync with a multicentric study conducted in 2009 in West Bengal, India [13], where the male:female ratio was 21:17. A three-year retrospective study done in China from 2009 to 2011 [14] shows a male-to-female ratio between 1.69:1 and 1.85:1. Our study revealed a variation in HFMD cases, with peaks occurring during the summer and early autumn months of August and September. This pattern aligns with findings reported in Kerala [15] and China [16], suggesting a consistent epidemiological trend across different geographical regions.

Regarding clinical manifestations, vesiculopapular lesions on the hands and feet were the most predominant symptom observed in our study and were seen in 100% of the HFMD cases, followed by lesions on the buttocks (91.96%) and elbows (48.21%). This is consistent with findings reported in Shimoga City, Karnataka, India [17], where the most common presentation was vesicles on the hands or feet which were present in all the cases diagnosed, followed by oral ulcers (89%) and vesicles on the buttocks (65%). In our study conducted in Jammu, we found that 71% of cases presented with oral lesions in the form of aphthous ulcers, while 15.68% had oral lesions consistent with stomatitis. A study conducted in Kolkata, India [18] shows that the palate (51.6%) was the most common site of intraoral involvement, followed by the buccal mucosa (24.2%), tongue (21%), anterior pillar of tonsils (17.7%), gingiva (9.7%), and lips (6.4%).

In the present study, painful deglutition (63.39%) was found to be the most commonly associated constitutional symptom, followed by fever (57.14%), loss of appetite (42.86%), lethargy and irritability (41.96%), pruritis (21.43%), sore throat (9.82%), and dehydration (7.14%) in decreasing order. When comparing our findings with those of Agarwal in Udaipur, Rajasthan [19] and Sabitha S in Kerala, India [20], differences in the prevalence of constitutional symptoms become apparent. Agarwal reported a higher prevalence of fever (78.3%) and painful deglutition (89%) compared to our study, while lethargy/irritability (56.34%) and pruritis (43.45%) were also more prevalent in their study. Conversely, Sabitha S reported a similar prevalence of fever (65.67%) but lower rates of lethargy/irritability (39%) and painful deglutition (32.67%) compared to our study. These variations in constitutional symptoms across different regions may reflect differences in the virulence of circulating viral strains, as well as variations in population demographics and healthcare-seeking behavior. Healthcare providers should be aware of these variations in constitutional symptoms to ensure accurate diagnosis and appropriate management of HFMD cases, particularly in regions where certain symptoms are more prevalent.

Additionally, in this study, we also observed that in n=31 cases (27.68%), a history of contact was seen where the disease was present in another child of the same family currently or had HFMD infection in the past, suggesting the potential of person-to-person transmission within close contacts. The role of history of contact has also been highlighted in a study conducted by Jiratchya [21], which emphasizes the fact that in almost 10% of cases, HFMD is acquired due to the history of contact with a sibling or other child member of the family who already has HFMD or has recently recovered from the disease. This study also mentions the role of a clean environment and hygiene in preventing HFMD. Furthermore, in the present study, nail changes in the form of onychomadesis, Beau's lines, and yellowish-orange discoloration of the nail were observed in a few cases (1.79%) during follow-ups, highlighting potential post-recovery complications associated with HFMD. Nail changes during follow-up were also seen in studies done by Verma in New Delhi, India [22] and Alghamdi in Jeddah, Saudi Arabia [23]. Healthcare providers should be aware of these nail changes to better understand the complications associated with HFMD. In summary, our study findings align with previous research conducted in different regions of India, providing valuable insights into the age distribution and clinical characteristics of HFMD. These findings underscore the importance of ongoing surveillance and public health interventions to mitigate the burden of HFMD in pediatric populations across diverse geographical settings.

Limitations of the study

While our study provides valuable insights into the epidemiology and clinical characteristics of hand, foot, and mouth disease (HFMD), several limitations warrant consideration. The study's geographical restriction to a specific region may limit the generalizability of our findings to broader populations with different demographic and environmental profiles. Variability in diagnostic practices across healthcare settings may further impact the consistency and reliability of our results. Moreover, the sample size limitations and lack of long-term follow-up data constrain our ability to comprehensively assess clinical outcomes and post-recovery complications. Future multicentric studies with larger cohorts and standardized methodologies are essential to address these limitations and enhance our understanding of HFMD epidemiology and management.

## Conclusions

Although HFMD is typically not a serious illness, it is highly contagious. It can spread quickly at preschool and daycare centers; therefore, more awareness programs should be conducted to educate parents and promote hygiene to prevent contact cases. Surveillance programs are needed to identify the causes of the seasonal spike. Healthcare providers and the medical community can play a vital role in enhancing public awareness to decrease and prevent the incidence of future HFMD cases and outbreaks.
